# Case Report: A delicate equilibrium of exocrine pancreatic recovery and hepatotoxicity with elexacaftor/tezacaftor/ivacaftor therapy in a pediatric patient with cystic fibrosis

**DOI:** 10.3389/fped.2024.1457517

**Published:** 2024-10-23

**Authors:** Michael P. Coughlin, Senthilkumar Sankararaman, Erica A. Roesch, Emily D. Certo, Benjamin L. Brej, Michael W. Konstan

**Affiliations:** ^1^ Department of Pediatrics, Division of Pediatric Pulmonology, Rainbow Babies & Children’s Hospital, Cleveland, OH, United States; ^2^ Department of Pediatrics, Division of Pediatric Gastroenterology, Rainbow Babies & Children’s Hospital, Cleveland, OH, United States; ^3^ Department of Pediatrics, Case Western Reserve University School of Medicine, Cleveland, OH, United States; ^4^ Department of Pediatrics, Rainbow Babies & Children’s Hospital, Cleveland, OH, United States; ^5^ The Ohio State University College of Medicine, Columbus, OH, United States

**Keywords:** case report, cystic fibrosis (CF), cystic fibrosis transmembrane conductance regulator (CFTR) modulators, elexacaftor/tezacaftor/ivacaftor (ETI), exocrine pancreatic function, hepatotoxicity

## Abstract

This case report presents a comprehensive evaluation of the complex balance of therapeutic benefits and potential risks associated with the cystic fibrosis transmembrane conductance regulator (CFTR) modulator elexacaftor/tezacaftor/ivacaftor (ETI) therapy in managing an eight-year-old male with cystic fibrosis (CF) and exocrine pancreatic insufficiency (EPI). While ETI therapy significantly enhanced exocrine pancreatic function, it led to hepatotoxicity, necessitating therapy discontinuation. Attempts to restart ETI at reduced doses were unsuccessful due to persistent hepatic dysfunction. Reduced ETI dosing frequency, implemented due to hepatic dysfunctions, did not result in substantial therapeutic benefits. Clinical markers showed a resurgence of severe EPI and sustained need for gastrostomy tube feeds, with only modest improvement in hepatic function compared to the period following ETI cessation or during prior use of CFTR modulator therapy with lumacaftor/ivacaftor. This case underscores the importance of personalized therapeutic approaches, biomarker-guided monitoring, and multidisciplinary insights to optimize CF management while also highlighting the ongoing need for research to mitigate hepatotoxicity risks and ensure long-term therapeutic efficacy.

## Introduction

Cystic fibrosis (CF) is a prevalent autosomal-recessive disorder caused by mutations in the cystic fibrosis transmembrane conductance regulator (CFTR) gene, affecting more than 30,000 individuals in the United States and over 160,000 globally, based on estimates (1). CF has multi-organ implications, particularly impacting the respiratory, digestive, and hepatic systems due to absent or defective CFTR protein function (2). Approximately 85% of people with CF develop exocrine pancreatic insufficiency (EPI) at an early age, accounting for significant morbidity (3). Poorly controlled EPI leads to malnutrition with poor weight gain, impacting pulmonary function and overall health outcomes (4). Fecal pancreatic elastase-1 (FE-1) measurement is the most widely used indirect test to assess EPI, with values less than 200 µg/g of stool consistent with EPI (5).

Recent advances in CF care have dramatically transformed both the diagnosis and treatment of the disease. Newborn screening has been crucial in revolutionizing CF diagnosis, allowing for pre-symptomatic early detection and alleviating the distressing diagnostic journeys that many patients and families previously experienced (6). Early diagnosis enables timely interventions that can mitigate disease progression from infancy, leading to improved clinical outcomes (6). On the treatment side, significant advancements have been the development of CFTR modulator therapies that directly target defective CFTR (7). These CFTR therapies enhance chloride ion transport across epithelial surfaces, including in the pancreatic ducts, which may ameliorate EPI signs and symptoms (8). CFTR modulator therapy has made it possible for some people with CF to experience improvement in FE-1, suggesting an improvement in their exocrine pancreatic function. Recent studies have shown that the CFTR modulator elexacaftor/tezacaftor/ivacaftor (ETI) can enhance and restore exocrine pancreatic function in children with CF, as evidenced by changes in FE-1 levels (9–12).

While these modulators, such as ETI, show promise in restoring pancreatic function, they also carry risks of hepatotoxicity (13). This case report examines the complexities of ETI therapy in managing a pediatric patient with CF, focusing on the balance between improved pancreatic function and hepatotoxicity. The findings highlight the need for patient-specific treatment strategies and provide insights into the complexities of CFTR modulators, emphasizing the careful balance between therapeutic benefits and potential risks.

## Case description

Our patient is an eight-year-old male with CF. At birth, the newborn screen revealed elevated immunoreactive trypsinogen and genetic testing confirmed two pathogenic CF mutations in the CFTR gene (F508del/F508del). Confirmatory sweat chloride testing revealed levels of 109 and 118 mmol/L (average 113.5 mmol/L), well above the diagnostic threshold for CF of ≥60 mmol/L. Pancreatic enzyme replacement therapy and fat-soluble vitamins were initiated at diagnosis. At two weeks of age, FE-1 testing showed EPI with a level of 55 µg/g stool (correlating with severe pancreatic insufficiency at <100 µg/g of stool). At seven months of age, FE-1 testing was repeated, again revealing severe EPI (19 µg/g). At one year of age, gastrostomy tube (G-tube) feeds were initiated to improve the patient's nutritional status.

At three years of age, the patient began the CFTR modulator lumacaftor/ivacaftor (LUM/IVA) therapy and continued to require G-tube feeds until six years of age. These feeds were discontinued following a significant increase in weight and BMI percentages. A few months later, the patient transitioned from LUM/IVA to ETI. Despite an initial subjective improvement in appetite, the patient experienced a sharp decline in weight and BMI percentiles after stopping G-tube feeds and initiating ETI, leading to reinitiation of G-tube feeds. This intervention was followed by subsequent weight gain of 2 kg over two months, which once again led to the discontinuation of G-tube feeds. Overall, fluctuations in weight and BMI were effectively managed through G-tube feeds.

After nine months of ETI therapy, FE-1 improved to 233 µg/g, correlating with exocrine pancreatic sufficiency (defined as a level >200 µg/g). This status was confirmed three months later, with a value of 288 µg/g. Concurrently, the patient had hepatic function abnormalities, with an elevation of alanine aminotransferase (ALT) of 110 U/L (>3× the upper limit of normal (ULN)) and total bilirubin of 1.4 mg/dl (>2× ULN), prompting discontinuation of ETI as per prescribing information guidelines. These guidelines state that if ALT or aspartate aminotransferase (AST) levels are >5× ULN, or if ALT or AST levels are >3× ULN with bilirubin levels >2× ULN, dosing should be interrupted and tests closely monitored until resolved (14). Of note, liver function tests were normal prior to the initiation of both CFTR modulator therapies. A hepatic ultrasound was performed following hepatic function abnormalities, and the results were normal.

Three months after discontinuing ETI, FE-1 decreased to <10 µg/g, consistent with severe EPI. The full therapeutic ETI dose could not be restarted due to persistent hepatic function abnormalities. Instead, reduced dosing regimens of ETI were attempted with ongoing assessments of clinical outcomes and medication-induced adverse effects. Despite these changes, the hepatic function abnormalities persisted. As a result, ETI was discontinued completely, leading to the normalization of hepatic function labs. The patient was then successfully restarted on LUM/IVA, a regimen previously well-tolerated without hepatic complications, and has since continued to tolerate that treatment without any issues.

Throughout the patient's life, routine annual testing for fat-soluble vitamins has been conducted, with supplementation adjusted as needed to maintain levels within the normal range. The patient has a history of *Pseudomonas aeruginosa* growth on CF throat cultures, first at one year of age and again at four years of age, both without significant clinical impact or need for hospitalization. Both instances were appropriately treated with inhaled tobramycin and there has been no recurrence. Typically, the patient's CF throat cultures, obtained approximately four times per year, are normal. The patient has had sporadic growth of methicillin-sensitive *Staphylococcus aureus* and *Haemophilus influenzae* in the past, but not while on ETI. Throughout life, the patient has had no significant pulmonary exacerbations requiring hospitalization or intravenous antibiotics.

## Diagnostic assessment

**Figure 1:**
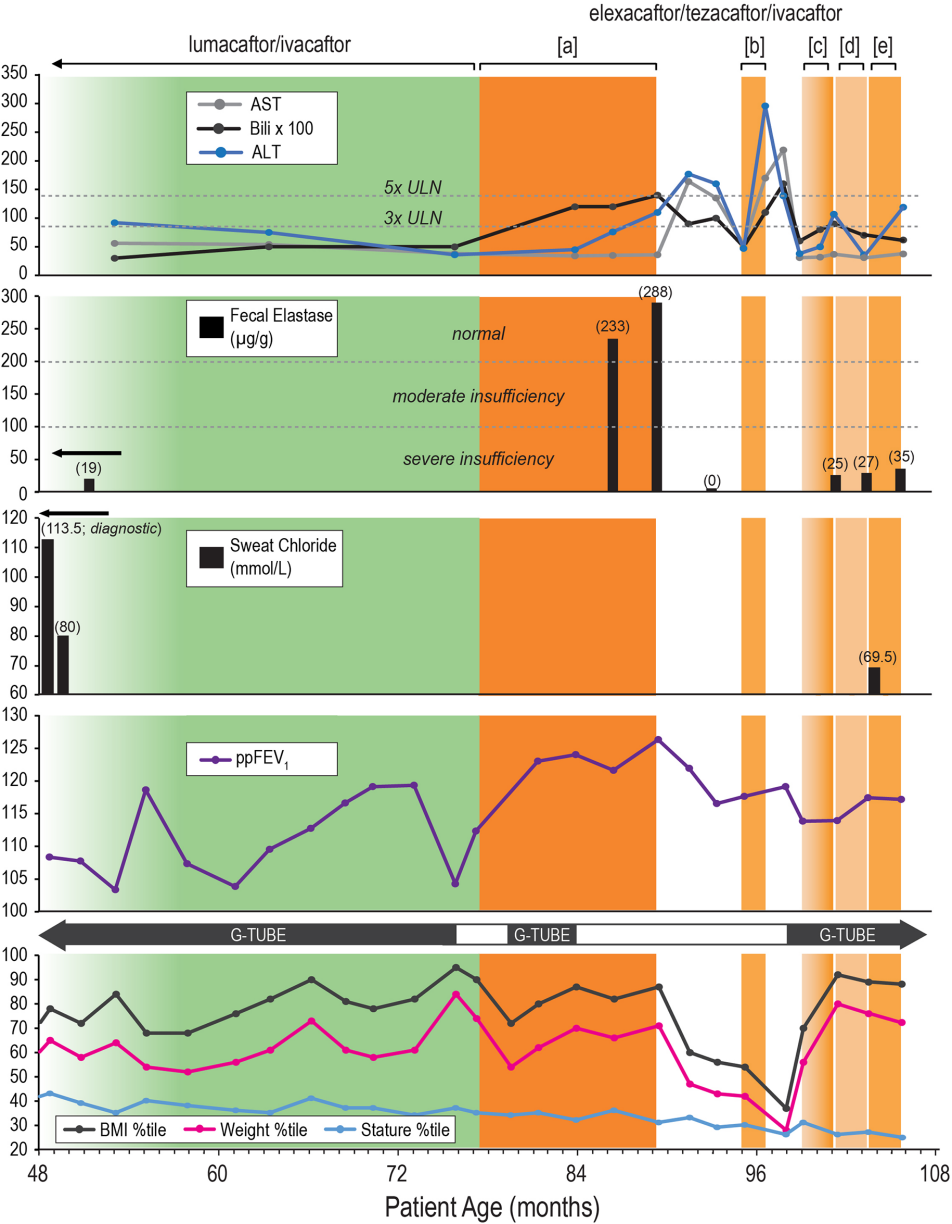
Clinical course and management timeline of an eight-year-old male with cystic fibrosis. **Description:** Clinical course and management of an eight-year-old male with cystic fibrosis (CF) and exocrine pancreatic insufficiency (EPI) from age 4–8 years (expressed in months of age), highlighting key interventions and outcomes (clinical markers) related to cystic fibrosis transmembrane conductance regulator (CFTR) modulator therapies. **Therapeutic Interventions:** lumacaftor/ivacaftor (LUM/IVA): green-shaded area. Elexacaftor/tezacaftor/ivacaftor (ETI): Orange-shaded areas with different dosing regimens: [a]: Full dose (daily), [b]: Partial dose (4 days/week), [c]: Ramp-up dosing (2 days/week) → (3 days/week) → (4 days/week), [d]: Partial dose (3 days/week), [e]: Partial dose (4 days/week). ETI Discontinuation Periods: White area. **Outcome Panels:** Liver Function Tests: Alanine Aminotransferase (ALT): Blue line. Aspartate Aminotransferase (AST): Gray line. Bilirubin: Black line, multiplied by 100 for visualization. Normal and elevated thresholds (3x ULN and 5x ULN) are marked. Significant elevations in ALT and bilirubin during ETI therapy leading to therapy discontinuation. Fecal Elastase (FE-1): Severe insufficiency (< 100 µg/g), moderate insufficiency (100–200 µg/g), and normal (> 200 µg/g) ranges indicated. Initial severe insufficiency (19 µg/g) improved to normal levels (233 and 288 µg/g) on ETI therapy, followed by a decline (< 10 µg/g as represented by 0 µg/g) after therapy discontinuation. While on partial dose ETI regimens, the patient’s FE-1 levels remained in the severe insufficiency range, with values of 25, 27, and 35 µg/g. Sweat Chloride: Diagnostic and follow-up levels, with initial high levels indicating CF (113.5 mmol/L, reflecting the average of right and left arm values), improved levels on LUM/IVA (80 mmol/L), and during ETI therapy (69.5 mmol/L). Percent Predicted Forced Expiratory Volume in 1 second (ppFEV1): Mild fluctuations in lung function over time with a general stable trend. Growth Metrics with Gastrostomy Tube (G-tube) Use: Body Mass Index (BMI) Percentile: Black line. Weight Percentile: Pink line. Stature Percentile: Blue line. Periods of G-tube use indicated with black bars, showing changes in dependency related to nutritional status and therapy. The patient continued to depend on G-tube feeding for appropriate weight gain on a varied reduced ETI dosing frequency. **Overall Findings:** Reduced ETI dosing frequency, implemented due to hepatic dysfunctions, did not result in substantial therapeutic benefits. On reduced ETI dosing frequency, clinical markers showed a resurgence of severe EPI and sustained need for G-tube feeds, with only modest improvement in hepatic function compared to cessation of ETI or prior therapy with LUM/IVA.

Figure 1 illustrates our patient's clinical course and management from age 4–8 years (expressed in months of age), highlighting key interventions and outcomes (clinical markers) related to CFTR modulator therapies. We monitored FE-1 levels, sweat chloride levels, spirometry [percentage predicted forced expiratory volume per second (ppFEV1)], anthropometric measurements, and the need for G-tube feeds. At the same time, hepatic function was evaluated to determine tolerance to the modified dosing regimen.

Analysis of the patient data presented in Figure 1 revealed that the reduced ETI dosing frequency, implemented in response to hepatic dysfunctions, was not associated with substantial therapeutic benefits. Clinical markers indicated a resurgence of severe EPI. Furthermore, the reduced ETI dosing frequency led to only a modest improvement in hepatic function, which was less significant compared to the cessation of ETI or the effects observed during prior therapy with LUM/IVA.

The anthropometric data and the need for G-tube feeds presented in Figure 1 suggest that G-tube feeds are closely linked to sustained weight and BMI percentiles rather than to CFTR modulator therapy. This is especially clear following the 76-month age mark, where the patient experienced a sharp decline in weight (from the 84th to the 54th percentile) and BMI percentiles (from the 95th to the 72nd percentile) after stopping G-tube feeds, despite the initiation of full-dose ETI. This decline led to the reinitiation of G-tube feeds, which was followed by improvements in weight to the 70th percentile and BMI to the 87th percentile, which again led to the discontinuation of G-tube feeds.

Sweat chloride testing revealed important information about how the patient responded to CFTR modulators. As seen in Figure 1, both LUM/IVA and lower-dose ETI regimens showed improved sweat chloride levels (80 and 69.5 mmol/L, respectively), suggesting a degree of CFTR modulation. Unfortunately, sweat chloride levels were not obtained on full-dose ETI.

The final clinical marker not yet mentioned in Figure 1 is ppFEV1, which showed mild fluctuations throughout all therapeutic interventions, with a generally stable and reassuring trend. The average lifetime ppFEV1 is 115%.

## Discussion

### Overview of findings

This case report describes an eight-year-old male with CF and EPI who experienced significant improvements in pancreatic function with ETI therapy but also developed hepatotoxicity, leading to therapy discontinuation.

### Comparison with existing literature

CFTR modulators like ivacaftor have been shown to improve pancreatic function in children with CF. The phase 3 KIWI study and its extension, the KLIMB study, reported significant increases in mean FE-1 levels by 99.8 µg/g in children aged 2–5 years with gating mutations (9, 10). Additionally, a study on ETI therapy in children with CF (F508del homozygous) aged 6–11 years found that ETI significantly improved pancreatic function, increasing FE-1 levels by an average of 140 µg/g (11). Similarly, Stephenson et al. observed that younger children (<10 years) with CF (F508del homozygous) had the greatest improvements in FE-1 levels when treated with ETI (12).

Our patient is homozygous for the F508del mutation, which is commonly associated with severe EPI (3). Our case aligns with previous reports in patients of similar age and mutation status as described above, showing the patient's transition from severe pancreatic insufficiency to sufficiency (FE-1 levels >200 µg/g) on ETI therapy. However, the PROMISE study, a large 56-center prospective observational study in people with CF aged 12 years and older with at least one F508del allele, found that ETI therapy did not result in sustained improvement in pancreatic function in older children. Among the 99 participants (38 between the ages of 12 and 18 years) with data from both baseline and six months, no significant improvement in FE-1 levels was seen. The PROMISE study anticipated these findings, highlighting that EPI, which often begins *in utero*, becomes increasingly difficult to reverse as the disease progresses. It suggested that reversing long-standing EPI in a fibrotic or fatty-replaced pancreas is unlikely, even with effective CFTR modulation (15).

### Hepatotoxicity and Its management

Our case highlights a significant adverse effect: hepatotoxicity. This aligns with clinical trial data indicating the need for careful hepatic monitoring due to the risk of elevated liver enzymes. In a phase 3 clinical trial of ETI in 66 children aged 6–11 years with at least one F508del allele, 10.6% showed elevated ALT/AST levels >3× ULN, with 1.5% >5× ULN. No children had AST >3× ULN along with bilirubin >2× ULN. There were no interruptions or discontinuation of ETI therapy due to elevated liver enzymes (13).

The exact cause of ETI-induced hepatotoxicity remains unclear, but it is thought to involve the generation of toxic or immune metabolites that affect the cytochrome p450 system, mainly the CYP 3A (16). Given this, caution is advised when using ETI in combination with medications that inhibit the CYP 3A system as these interactions can increase the risk of liver injury (16).

Tewkesbury et al. noted the multifactorial nature of elevated liver enzymes in patients with CF on ETI, noting that many cases could be linked to other causes such as viral infections or pre-existing liver conditions. Their findings emphasize the importance of thorough evaluation to exclude other factors before attributing hepatotoxicity to ETI (17). In cases of suspected drug-induced liver injury (DILI), close monitoring and involvement of hepatology specialists are crucial for deciding whether ETI therapy can be safely reintroduced or modified (17).

A recent study using the FDA adverse event reporting system found that most cases of ETI-induced liver injury occurred within three months of initiation, with a median onset time of 50.5 days (18). Depending on the severity of liver injury, treatment may require discontinuation or dose reduction, though the risk of recurrence upon reintroduction remains unpredictable (18).

Unlike our case, where the reintroduction of ETI was not possible, Stylemans et al. successfully reintroduced ETI at a lower dose after an initial episode of DILI, highlighting the unpredictable nature of this adverse effect (19).

### ETI tolerance in post-liver transplant patients

Another important consideration is the safety of ETI therapy in patients with CF who have undergone liver transplantation due to CF-related liver disease progressing to failure. A systematic review by Testa et al. evaluated 20 post-liver transplant patients with CF, aged 14–45 years. This review found that while ETI was generally well-tolerated, liver function abnormalities, particularly elevated transaminases, were observed in several cases. Specifically, five patients required dose modifications due to these elevations, necessitating temporary discontinuation or dose reduction. Of these, three patients tolerated ETI after resuming at a reduced dose, while one patient managed to continue at the full dose (20).

### Sweat chloride as a marker of CFTR modulation

Sweat chloride testing provided insights into the patient's response to CFTR modulators. Both LUM/IVA and lower-dose ETI regimens showed improved sweat chloride levels, suggesting a degree of CFTR modulation. However, the sweat chloride levels observed with reduced ETI dosing frequency did not achieve the same reduction seen with the standard ETI dosing. This is compared to sweat chloride data in children with CF (F508del homozygous), which showed a median post-ETI sweat chloride level of 42 mmol/L for the 6–11 years age group (21). This suggests sub-optimal dose efficacy for our patient with a sweat chloride of 69.5 mmol/L on reduced ETI dosing frequency.

### Unique aspects and novel insights

The complete rebound of pancreatic function in our patient, despite pre-existing severe EPI, suggests that early intervention with CFTR modulators might alter the natural course of pancreatic disease in CF, a concept previously thought unattainable. However, the re-emergence of severe EPI upon discontinuation of ETI due to hepatotoxicity underscores the need for alternative strategies to manage adverse effects.

### Clinical implications

The complex balance of ETI therapy in our patient, where pancreatic function was restored but hepatic function was compromised, emphasizes the importance of personalized therapeutic strategies and vigilant monitoring. Regular hepatic function tests are crucial when administering ETI to mitigate risks and manage potential hepatotoxicity. Additionally, reduced ETI dosing frequency, implemented due to hepatic dysfunctions, did not result in substantial therapeutic benefits. This finding underscores that simply reducing the dosage to manage side effects may not be effective, stressing the need for alternative strategies or additional interventions to maintain therapeutic efficacy while minimizing adverse effects.

Given the patient's previous tolerance to LUM/IVA without hepatic abnormalities, we elected to restart this regimen to maintain CFTR modulation. Looking ahead, vanzacaftor/tezacaftor/deutivacaftor presents a potential treatment option for the patient, pending regulatory approval (22). However, it remains uncertain how the patient will tolerate this combination therapy, especially given the previous intolerance to ETI, which also contains tezacaftor.

### Role of nutritional support and monitoring

This case highlights the importance of nutritional support, specifically G-tube feeds, in maintaining weight and BMI even when on CFTR modulator therapy. The growth data shows that G-tube feeds were the primary driver of sustained weight and BMI rather than CFTR modulators. These findings emphasize the need for providers to be closely aware of monitoring outcomes related to CFTR modulator therapies, such as anthropometric data, while considering the effects of other clinical interventions like G-tube feeds. It is essential to clearly distinguish the contributions of each intervention to avoid confounding the overall treatment outcomes.

## Conclusion

This case report sheds light on the delicate equilibrium of therapeutic benefits and potential risks of ETI therapy in CF management, showing its potential to restore pancreatic function while posing risks of hepatic complications. The findings emphasize the importance of careful monitoring and thoughtful application of ETI, especially regarding dose adjustments. The clinical course underscores the need for rigorous hepatic monitoring, establishing clear thresholds and optimal assessment duration to ensure patient safety and therapeutic effectiveness.

Future research should focus on developing CFTR modulators with improved safety profiles to enhance CF care. Understanding the mechanisms of ETI-induced hepatotoxicity is crucial for enhancing treatment outcomes and minimizing adverse effects. This case contributes to the deeper understanding of CFTR therapies and encourages further investigation into their optimal use, balancing efficacy and safety to improve the lives of those with CF.

## Data Availability

The original contributions presented in the study are included in the article/Supplementary Material, further inquiries can be directed to the corresponding authors.
